# Diagnosis and Management of Pediatric Ear and Vestibular Disorders

**DOI:** 10.3390/children13060833

**Published:** 2026-06-19

**Authors:** Mirko Aldè, Stefania Barozzi

**Affiliations:** 1 Department of Clinical Sciences and Community Health, Department of Excellence 2023–2027, University of Milan, 20122 Milan, Italy; stefania.barozzi@unimi.it; 2 Audiology Unit, Department of Specialist Surgical Sciences, Fondazione IRCCS Ca’ Granda Ospedale Maggiore Policlinico, 20122 Milan, Italy

Hearing and balance disorders represent some of the most challenging conditions encountered in pediatric healthcare [1]. Although substantial progress has been achieved in newborn hearing screening, audiological assessment, and otologic surgery over recent decades, many children with auditory and vestibular dysfunction continue to experience delayed diagnosis, limited access to specialized care, and significant impacts on communication, learning, motor development, and quality of life [2,3]. The complexity of these disorders is amplified by the unique developmental characteristics of childhood, which often make symptom recognition and clinical evaluation more difficult than in adult populations [4].

The Special Issue “*Diagnosis and Management of Pediatric Ear and Vestibular Disorders*” was designed to highlight current challenges and emerging opportunities in this important yet still understudied field, with contributions addressing the diagnosis of vestibular disorders, the diagnosis and treatment of hearing loss (HL), innovative rehabilitation approaches such as telerehabilitation and music-based interventions, and the management of complications related to otitis media. Overall, nine original articles and one review are included in this Special Issue, as listed below:

1. Božanić Urbančič, N.; Mladenov, D.; Battelino, S. The Impact of Follow-Up on Etiological Classification of Pediatric Vertigo. *Children* **2026**, *13*, 376. https://doi.org/10.3390/children13030376
2. Norton, C.P.; Darr, N.S.; Beshears, M.K.; Catalano, K.; Dillard, T.; Gamble, M.J.K.; Olerich, M.; Rupp, S.R. Non-Instrumented DVA: Assessment of Performance and Clinical Feasibility in Children Ages 2 Through 13 Years. *Children* **2026**, *13*, 456. https://doi.org/10.3390/children13040456

Specifically, it was shown that longitudinal vestibular follow-up should be regarded as an essential component of the diagnostic pathway, facilitating greater diagnostic accuracy and a more comprehensive understanding of the underlying disorder. Alongside this, the feasibility of non-instrumented dynamic visual acuity (DVA) testing in children was demonstrated, supporting the use of a simple and accessible screening tool that may facilitate the early identification of vestibular dysfunction in routine clinical practice.

The studies on auditory disorders mainly focused on various aspects of diagnosis, rehabilitation, and treatment, and the contributions are listed below:

1. Brotto, D.; Impalà, G.; Lovato, E.; Mazzaro, E.; Maculan, M.; Zanoletti, E.; Galoforo, N.; Trevisi, P. ABR Features in Ski-Slope Hearing Loss for Hearing Threshold Estimation: A Comparative Clinical Study of Click and CE-Chirp Stimuli. *Children* **2026**, *13*, 410. https://doi.org/10.3390/children13030410.
2. Aldè, M.; Zanetti, D.; Ambrosetti, U.; Monaco, E.; Gasbarre, A.M.; Pignataro, L.; Cantarella, G.; Barozzi, S. Unilateral Sensorineural Hearing Loss in Children: Etiology, Audiological Characteristics, and Treatment. *Children* **2024**, *11*, 324. https://doi.org/10.3390/children11030324.
3. Lauriello, M.; Angelone, A.M.; Iannotti, S.; Nardecchia, E.; Scopano, B.; Fioretti, A.; Ciancarelli, I.; Eibenstein, A. Audiophonologopedic Telerehabilitation: Advantages and Disadvantages from User Perspectives. *Children* **2024**, *11*, 1073. https://doi.org/10.3390/children11091073.
4. Aldè, M.; Casella, L.; Ambrosetti, U.; Barozzi, S.; Gandolfo, E.; Di Berardino, F.; Zanetti, D. Music-Based Interventions in Childhood Hearing Loss: A Comprehensive Narrative Review. *Children* **2026**, *13*, 574. https://doi.org/10.3390/children13040574.

It was reported that, in the context of auditory brainstem responses (ABRs), CE-Chirp stimulation offers clearer advantages in threshold estimation and neural synchrony, particularly in pediatric patients with ski-sloping HL. Moreover, children with unilateral HL were shown to perform significantly better on audiological tests, including speech perception in quiet and noisy environments and sound localization, while using hearing devices. These findings suggest that greater attention should be devoted to the timely identification and rehabilitation of these patients. Finally, telerehabilitation and music-based interventions emerged as valuable tools for children with HL, representing feasible approaches for future rehabilitation strategies.

Another important topic addressed in this Special Issue was the management of complications of otitis media, with contributions exploring endoscopic myringoplasty, the pediatric utilization of ophthalmic antibiotics in the ear, and the clinical management of acute mastoiditis, as listed below:

1. Nocini, R.; Monzani, D.; Arietti, V.; Bonasera, F.; Bianconi, L.; Sacchetto, L. Endoscopic Myringoplasty for Pediatric Tympanic Membrane Perforations: Is It Worth It? *Children* **2025**, *12*, 293. https://doi.org/10.3390/children12030293.
2. Cheung, I.M.Y.; Yin, T.; Gokul, A. Paediatric Utilisation of Ophthalmic Antibiotics in the Ear in Aotearoa/New Zealand. *Children* **2025**, *12*, 557. https://doi.org/10.3390/children12050557.
3. Alshehri, S.; Alahmari, K.A. Pediatric Acute Mastoiditis in Saudi Arabia: Demographic Insights, Clinical Profiles, and Prognostic Factors. *Children* **2024**, *11*, 402. https://doi.org/10.3390/children11040402.
4. Sabitova, N.; Shamshudinov, T.; Kussainova, A.; Toguzbayeva, D.; Sadykov, B.; Rahanskaya, Y.; Kassym, L. A National Forecast and Clinical Analysis of Pediatric Acute Mastoiditis in Kazakhstan. *Children* **2026**, *13*, 170. https://doi.org/10.3390/children13020170.

Endoscopic myringoplasty was shown to be a safe procedure with a high success rate for the repair of tympanic membrane perforations, while further collaborative efforts are needed to optimize the appropriate use of ophthalmic ciprofloxacin for ear infections. The studies from Saudi Arabia and Kazakhstan demonstrated that pediatric acute mastoiditis is both a clinically significant complication of otitis media and a sensitive indicator of broader healthcare system constraints, underscoring the need for earlier diagnosis, standardized management pathways, and strengthened pediatric otolaryngology services to prevent severe complications and improve outcomes.

Overall, this Special Issue provides novel insights and has the potential to contribute significantly to the diagnosis, management, treatment, and rehabilitation of hearing and vestibular disorders in children. Future research should focus on the implementation of universal vestibular screening programs for children with HL [5], the development of more reliable diagnostic tools for vestibular disorders in early childhood [3,4], and the advancement of innovative hearing and vestibular rehabilitation strategies [6]. Particular attention should be devoted to ensuring that technologically advanced diagnostic and rehabilitative tools become accessible to pediatric populations worldwide [7].
